# Household Crowding, Social Mixing Patterns and Respiratory Symptoms in Seven Countries of the African Meningitis Belt

**DOI:** 10.1371/journal.pone.0101129

**Published:** 2014-07-02

**Authors:** Claire F. Ferraro, Caroline L. Trotter, Maria C. Nascimento, Jean-François Jusot, Babatunji A. Omotara, Abraham Hodgson, Oumer Ali, Serge Alavo, Samba Sow, Doumagoum Moto Daugla, James M. Stuart

**Affiliations:** 1 Department of Infectious & Tropical Diseases, London School of Hygiene & Tropical Medicine, London, United Kingdom; 2 Disease Dynamics Unit, Department of Veterinary Medicine, University of Cambridge, Cambridge, United Kingdom; 3 Unité d'Epidémiologie, Centre de Recherches Médicales et Sanitaires (CERMES), Niamey, Niger; 4 Department of Community Medicine, University of Maiduguri, Maiduguri, Nigeria; 5 Navrongo Health Research Centre, Navrongo, Ghana; 6 Research and Development Division, Ghana Health Service, Ghana; 7 Armauer Hansen Research Institute, Addis Ababa, Ethiopia; 8 L'institut de recherche pour le développement, Dakar, Senegal; 9 Center for Vaccine Development-Mali (CVD-MALI), Bamako, Mali; 10 Centre de Support en Santé Internationale (CSSI), Ndjamena, Chad; Oxford University, Viet Nam

## Abstract

**Objectives:**

To describe the variation in household crowding and social mixing patterns in the African meningitis belt and to assess any association with self-reported recent respiratory symptoms.

**Methods:**

In 2010, the African Meningococcal Carriage Consortium (MenAfriCar) conducted cross-sectional surveys in urban and rural areas of seven countries. The number of household members, rooms per household, attendance at social gatherings and meeting places were recorded. Associations with self-reported recent respiratory symptoms were analysed by univariate and multivariate regression models.

**Results:**

The geometric mean people per room ranged from 1.9 to 2.8 between Ghana and Ethiopia respectively. Attendance at different types of social gatherings was variable by country, ranging from 0.5 to 1.5 per week. Those who attended 3 or more different types of social gatherings a week (frequent mixers) were more likely to be older, male (OR 1.27, p<0.001) and live in urban areas (OR 1.45, p<0.001). Frequent mixing and young age, but not increased household crowding, were associated with higher odds of self-reported respiratory symptoms (aOR 2.2, p<0.001 and OR 2.8, p<0.001 respectively). A limitation is that we did not measure school and workplace attendance.

**Conclusion:**

There are substantial variations in household crowding and social mixing patterns across the African meningitis belt. This study finds a clear association between age, increased social mixing and respiratory symptoms. It lays the foundation for designing and implementing more detailed studies of social contact patterns in this region.

## Introduction

Social mixing and human contact patterns are important determinants of infectious disease transmission. Household contacts are intensive and household crowding is a recognized risk factor for tuberculosis [1], [2] and acute rheumatic fever [3] in observational studies. Mass gatherings, such as the Hajj pilgrimage in Saudi Arabia, increase the risk of large-scale transmission and dissemination of infectious diseases, as seen with *Neisseria meningitidis* group W in 2001 [4]. Information on contact patterns is important for understanding the spread of infection and such information can be used, for example, to more accurately parameterise transmission models of infectious disease [5], [6]. However, information on mixing patterns and other social parameters are often lacking. There have been advances in collecting empirical social contact data by self-reported paper and electronic diaries [5], [7]–[9] or recall interviews [10] in Europe, Australia and Vietnam. To our knowledge, only one such similar study has been performed in sub-Saharan Africa [11]. Computational approaches have also been proposed recently to infer contact patterns in a population from sociodemographic data [12]–[14].

The African Meningococcal Carriage Consortium (www.menafricar.org) is a global research consortium that was established to study how meningococcal meningitis is spread in Africa [15] and to document the impact of a new serogroup A meningococcal conjugate vaccine [16] on reducing transmission. The consortium includes research institutes from seven countries across the African meningitis belt [17]; Chad, Ethiopia, Ghana, Mali, Niger, Nigeria and Senegal, which have diverse cultural, climatic, socio-demographic and epidemiological profiles. Previous meningococcal carriage studies in Africa have shown wide variation in prevalence ranging from less than 1% to over 30% [18]. Age patterns vary too, with the highest prevalence observed in children aged 1–4 years in Mali and Northern Nigeria, children <10years of age in Nigeria and teenagers in Northern Ghana and Burkina Faso [18], [19]. In the UK, meningococcal carriage in teenagers was particularly associated with social behaviour [20], so this variation in age distribution may represent diverse behavioural and social mixing patterns across the African region [18].

The MenAfriCar studies collected information on household size, composition and crowding in addition to data on attendance at social events and large gatherings such as the Hajj pilgrimage via standardized questionnaires. Everyday social activities such as school and workplace attendance were not measured. We set out to describe these household and social mixing patterns in the African meningitis belt and to examine associations between social and demographic factors with recent self-reported respiratory symptoms.

## Methods

### Study population

Household cross-sectional surveys were conducted in rural and urban areas of seven countries: Chad, Ethiopia, Ghana, Mali, Niger, Nigeria and Senegal, between 1st August and 30th December 2010, mainly during the rainy season. Households were randomly selected from a list derived from a demographic surveillance system (DSS) or, in sites without a DSS from a census performed specifically for the study. Following informed consent, the head of the household was interviewed using a short household questionnaire which recorded details relevant to the entire household such as number of residents, number of rooms, kitchen location and fuel type. Then, each randomly-selected individual resident within that household (up to 5 individuals per household, according to age) was interviewed using an adult (≥15 years) or child (<15 years) questionnaire, which collected individual level information, including attendance at social gatherings and sleeping arrangements. As the study was motivated by meningococcal carriage and not designed as a social mixing survey, we did not measure attendance at school and workplaces. Full details of the methods used in the MenAfriCar surveys are published elsewhere [15].

### Measures of risk factors, outcomes and potential confounders

Information on a range of factors including household size, household arrangements, social gathering attendance, kitchen location, fuel use and self-reported recent respiratory illness were collected in the cross-sectional surveys. Household crowding was summarised using a continuous variable called people-per-room, calculated by number of residents divided by number of rooms. The number of people per bedroom and people per bedmat were also used as additional crowding indicators.

Social mixing patterns were summarised according to the number of different types of social gatherings attended. Attending a market, wedding, funeral, ceremony (e.g. circumcision), social club, bar or other such event in the last week was considered a “social gathering”. The frequency of attendance and length of each social gathering within each of the sub-categories was not recorded. People were categorized by their social mixing patterns into *“non-mixers”* (individuals who attended no social gatherings in the last week), *“occasional mixers”* (individuals who attended one or two different types of social gatherings in the last week) and *“frequent mixers”* (individuals who attended 3–6 different types of social gatherings in the last week). Kitchen location and the types of cooking fuels used were recorded. Fuels were categorised according to biomass content (electricity; oil or gas or paraffin; wood or charcoal; animal dung or millet straws) (Fullerton et al., 2008) to indicate level of air pollution. The main outcome of interest was self-reported respiratory illness. This was defined as at least one of symptom (cough, sore-throat, or runny-nose) within the last week.

### Ethics

The MenAfriCar surveys were granted ethical approval by the London School of Hygiene & Tropical Medicine in May 2010. Each approval was also sought and gained from appropriate ethical committees of each country (AHRI-ALERT Ethics Review Committee, Ethiopia; Navrongo Heath Research Centre Institutional Review Board, Ghana; Ethics Committee of the Faculty of Medicine, University of Bamako, Mali; National Ethics Committee of Niger; Research and Ethics Committee of the University of Maiduguri Teaching Hospital, Nigeria; National Ethic Committee for Health Research, Senegal) with the exception of Chad, which did not have a formal ethical committee, where approval was granted by a committee set up to oversee MenAfriCar studies by the Ministry of Health. The study was registered with ClinicalTrials.gov (NCT01119482). Informed consent, as approved by the ethical committees, was obtained from participants as follows. The head of the household or another responsible adult gave verbal informed consent for the household to be included in the study. Each individual selected for inclusion within that household gave written informed consent (signature or thumbprint); for children under the age of 18 a parent or guardian gave written consent and children aged over 12 years were additionally asked to give written assent.

### Statistical methods

Logarithmic transformation was used to normalize skewed data (number of residents, rooms, bedrooms per household) and to present summary statistics of continuous variables by country, area, age and sex. Differences between populations in household crowding were evaluated by t-tests and chi-squared tests using data from household questionnaires only. Continuous explanatory variables (e.g. people per room, people per bedroom, people per bedmat) were categorized into four equal quartiles. Only data that were within 2 standard deviations (SD) of the mean for number of people per bedroom (99.0% were within 2SD) and bedmat (99.6% within 2SD) were included because the raw data included nonsensical values. Logistic regression was used to evaluate the characteristics associated with those people who mix more than others and risk factors associated with recent respiratory symptoms. First, univariate logistic regression was performed. Then a multivariable logistic regression model was constructed in a stepwise manner, with age and sex included *a priori*. Other variables associated with the outcome (p<0.1) in the univariate analysis were included, then any variables in the multivariable model with p<0.1 were excluded. Since the primary sampling unit for the survey was the household, rather than the individual, we adjusted for potential clustering within households by applying the svyset command to household id in Stata (v11.2, STATACorp, Texas). All statistical analyses were performed in Stata.

## Results

### Description of Data

MenAfriCar's first cross sectional study recruited and interviewed 18647 individuals across seven countries in the African Meningitis Belt. Combined household and individual data was available for 17805 (95.5%) individuals, (92.7% Mali, 99.6% Niger, 93.8% Ghana, 94.1% Nigeria, 98.4% Chad, 93.5% Ethiopia and 94.0% Senegal) from 6232 households. There were 8577 children aged <15 years (48.2%). There were more females than males overall (56.6% female, 43.4% male); the greatest sex difference existed in those aged 15–29 years old (67.3% female, 32.7% male). Household participation was good with 6% or fewer refusals in sites where this data was recorded.

### Household Size and Crowding

There was a large variation in household size (Table 1), with 1 to 90 residents reported per household (median 6; IQR 4,10), living in 1 to 62 rooms (median 3; IQR 2,5). (Note that the few extreme values reported were queried and verified with the local study teams). Household crowding, as measured by geometric mean people-per-room, also varied greatly (Table 1). Ghana and Senegal had a significantly lower geometric mean people-per-room compared to other countries, whilst Ethiopia had the most crowded households. There was no difference in crowding between rural and urban areas (geometric mean 2.14 and 2.20 respectively, p = 0.085).

**Table 1:** Summary of household data and crowding by country.

	Number of individuals recruited to study	Number of households recruited	Geometric mean of residents per household§	Geometric mean of people per room (95% CI)
Chad	2049	764	5.08	2.19 (2.09–2.30)
Ethiopia	1887	783	4.55	2.78 (2.66–2.91)
Ghana	1175	419	4.92	1.83 (1.73–1.94)
Mali	4662	1619	10.86	2.10 (2.05–2.16)
Niger	4642	1428	5.49	2.13 (2.07–2.19)
Nigeria	1758	653	6.22	2.31 (2.19–2.43)
Senegal	1632	566	9.14	1.87 (1.80–1.94)
**Total**	**17805**	**6232**	**6.68**	**2.17 (2.14**–**2.21)**

§ The geometric mean of residents per household is based on the number of individuals resident in the study households, and not the individuals recruited to the study shown in the second column above. (A maximum of 5 residents were recruited to the study).

### Social Mixing Patterns

The number of non-mixers, occasional mixers and frequent mixers, as previously defined, is shown in Table 2. Ghana had the highest proportion of frequent mixers and the lowest proportion of non-mixers, less than half any other country. Ghana also had the highest average number of types of social gathering per person, with Senegal reporting the lowest.

**Table 2:** Number (%) of individuals' social mixing patterns by country.

	N	Non-mixers N (%)	Occasional mixers N (%)	Frequent mixers N (%)	Number of different types of social gatherings per person*
Chad	2049	805 (39.3)	1128 (55.1)	116 (5.7)	0.91
Ethiopia	1887	1032 (54.7)	794 (42.1)	61 (3.2)	0.63
Ghana	1175	220 (18.7)	758 (64.5)	197 (16.8)	1.49
Mali	4662	2025 (43.4)	2455 (52.7)	182 (3.9)	0.79
Niger	4642	2227 (48.0)	1954 (42.1)	461 (9.9)	0.91
Nigeria	1758	957 (54.4)	707 (40.2)	94 (5.4)	0.68
Senegal	1632	948 (58.1)	665 (40.8)	19 (1.2)	0.53
**Total**	**17805**	**8214 (46.1)**	**8461 (47.5)**	**1130 (6.4)**	**0.83**

* Number of different types of social gatherings per person: total number of social gatherings in country/study population of that country.

Age was strongly associated with social mixing patterns (Table 3). When compared to older adults, 15–29 year olds had lower odds of being a frequent mixer (OR 0.63, p<0.001). The odds of being a frequent mixer were much lower for children aged less than 15 years (OR 0.29 for <1year olds, OR 0.13 for 1–4 year olds, OR 0.16 for 5–14 year olds, all p<0.001). In addition, frequent mixers had higher odds of living in urban areas (OR 1.45 p<0.001) and being male (OR 1.27 p<0.001).

**Table 3:** Multivariable analysis comparing frequent mixers to all others by age, sex and area.

	N	Number of (%) frequent mixers	Odds Ratio	(95% CI)		p-value
AGE						
<1 yr	869	34 (3.9)	0.29	(0.21	0.41)	<0.001
1–4 yr	3479	63 (1.8)	0.13	(0.10	0.17)	<0.001
5–14 yr	4230	94 (2.2)	0.16	(0.13	0.20)	<0.001
15–29 yr	4304	340 (7.9)	0.63	(0.55	0.72)	<0.001
>30 yr	4923	599 (12.2)	1			
SEX*						
Female	10030	619 (6.2)	1			
Male	7708	506 (6.3)	1.27	(1.11	1.44)	<0.001
AREA						
Rural	8510	442 (5.2)	1			
Urban	9295	688 (7.4)	1.45	(1.25	1.68)	<0.001

*missing data for 67 records.

In total across all countries, 165 adults had attended at least one large gathering in the last year (where a large gathering was classed as the Hajj or Umra pilgrimages, or other events with >10000 people). Attendance at large gatherings was associated with coming from an urban area (OR 2.50 p<0.001) but there was no difference between males and females (OR = 1.27 p = 0.144).

### Respiratory Symptoms in the last week

In the week preceding the survey, 8024 (45.5%) participants reported having at least one respiratory symptom. Runny nose was most commonly reported (36.3%), then cough (30.0%) and sore throat (7.0%). Factors associated with the reporting of respiratory symptoms in unvariate analyses included frequent mixing, i.e. attendance at 3 or more different types of social gatherings a week, younger age, separate or outdoor kitchen (Table 4). There was no association between fuel type and respiratory symptoms. There was also no linear association between people per room or bedroom and symptoms but an increase in the number of people sharing a bedmat increased the odds of having respiratory symptoms.

**Table 4:** Factors associated with reporting respiratory symptoms in the last week.

	N	N ≥1 symptom (%)	*Unadjusted OR* (95% CI)	p-value	*Adjusted OR* (95% CI)+	p-value
SOCIAL GATHERINGS						
Non-mixers	8214	3561 (43.4)	1		1	
Occasional mixers	8461	3913 (46.3)	1.12 (1.05, 1.20)	<0.001	1.37 (1.27, 1.47)	<0.001
Frequent mixers	1130	601(53.1)	1.48 (1.30, 1.69)	<0.001	2.25 (1.95, 2.61)	<0.001
NUMBER of PEOPLE PER ROOM						
0.125–1.6	4598	2116 (46.0%)	1		n/a	
1.6–2.33	4727	2101 (44.4%)	0.94 (0.85, 1.03)	0.203		
2.33–3.14	3969	1735 (43.7%)	0.91 (0.82, 1.01)	0.078		
3.14–17	4386	2081 (47.5%)	1.05 (0.95, 1.17)	0.276		
NUMBER of PEOPLE PER BEDROOM (cleaned <2sd)						
1–2	8461	3819 (45.1)	1		n/a	
3	3567	1712 (48.0)	1.12 (1.02, 1.22)	0.010		
4	2537	1142 (45.0)	0.99 (0.90, 1.10)	0.924		
>4	3010	1322 (43.9)	0.95 (0.86, 1.05)	0.330		
NUMBER of PEOPLE PER BEDMAT (cleaned <2sd)						
1	3811	1518 (41.5)	1		n/a	
2	8025	3588 (44.3)	1.12 (1.03, 1.22)	0.006		
3	4375	2155 (49.5)	1.37 (1.24, 1.50)	<0.001		
>3	1594	781 (49.0)	1.35 (1.19, 1.54)	<0.001		
KITCHEN LOCATION						
Inside house	4892	1994 (40.7)	1		1	
Open air	6729	3075 (45.7)	1.22 (1.11, 1.34)	<0.001	1.11 (0.99, 1.23)	0.056
Separate hut	5682	2824 (49.7)	1.44 (1.31, 1.58)	<0.001	1.05 (0.94, 1.18)	0.343
FUEL USE						
Electricity, Oil, Gas, Paraffin	267	133 (50.0)	1		n/a	
Wood, Charcoal	15559	7077 (45.5)	0.84 (0.65, 1.07)	0.169		
Animal Dung, Millet Straws	1747	786 (45.0)	0.82 (0.62, 1.07)	0.158		
AREA						
Rural	8510	4254 (50.0)	1		1	
Urban	9295	3821 (41.1)	0.70 (0.65, 0.75)	<0.001	0.71 (0.66, 0.77)	<0.001
AGE						
<1yr	869	557 (64.1)	2.72 (2.35, 3.15)	<0.001	3.17 (2.71, 3.70)	<0.001
0–4yr	3479	2083 (59.9)	2.28 (2.09, 2.48)	<0.001	2.79 (2.54, 3.07)	<0.001
5–14yr	4230	1816 (42.9)	1.14 (1.06, 1.24)	<0.001	1.30 (1.20, 1.42)	<0.001
15–29yr	4304	1671 (38.8)	0.97 (0.89, 1.05)	0.434	0.97 (0.90, 1.06)	0.555
>30yr	4923	1948 (45.3)	1		1	
SEX						
Female	10030	4505 (44.9)	1		1	
Male	7708	3534 (45.8)	1.04 (0.98, 1.10)	0.206	0.95 (0.89, 1.01)	0.116
SMOKING*						
No	8564	3355 (39.2)	1		n/a	
Yes	564	238 (42.2)	1.13 (0.95, 1.35)	0.16		
COUNTRY						
Chad	2049	1078 (52.6)	1		1	
Ethiopia	1887	910 (48.2)	0.84 (0.72, 0.97)	0.02	0.89 (0.75, 1.04)	0.162
Ghana	1175	622 (52.9)	1.01 (0.85, 1.20)	0.883	0.95 (0.79, 1,15)	0.590
Mali	4662	2443 (52.4)	0.99 (0.87, 1.12)	0.894	0.99 (0.87, 1.14)	0.916
Niger	4642	1658 (35.7)	0.50 (0.44, 0.57)	<0.001	0.52 (0.45, 0.60)	<0.001
Nigeria	1758	737 (41.9)	0.65 (0.56, 0.77)	<0.001	0.67 (0.57, 0.80)	<0.001
Senegal	1632	627 (38.4)	0.55 (0.47, 0.65)	<0.001	0.60 (0.49, 0.72)	<0.001

+ *The final multivariate logistic regression model included the following variables: country, social mixing, age, sex, area, kitchen location.*

*Only adults were questioned about their smoking habits.

A multivariate regression model found that social mixing patterns and age had the greatest impact on the odds of reporting respiratory symptoms (Table 4). Frequent mixers had higher odds of reporting respiratory symptoms than non-mixers (aOR 2.26, P<0.001). Adults had lower odds of respiratory symptoms, (test for trend of association with age in multivariate analysis: aOR = 0.73, p<0.001). The associations between reported respiratory symptoms and both the number of people per bedmat and location of the kitchen and symptoms were no longer evident.

## Discussion

This large and geographically extensive cross-sectional survey showed that household crowding is very common in seven countries of the African Meningitis Belt. The analysis of social mixing patterns of nearly 18000 individuals demonstrates large variations between countries and between urban and rural areas. In contrast to household crowding, social mixing patterns were associated with recent respiratory symptoms. The odds of reporting symptoms were more than two times higher in frequent mixers, a finding that was independent of age.

The number of residents per household in Ghana, Mali and Niger were comparable to other studies, supporting the evidence that on average there are larger households in Mali [21]–[23]. MenAfriCar surveys defined households as those who “share a cooking pot”, although much debate surrounds the most appropriate definition of households [24]. This is important as local norms of polygamy and extended family staying within the same compound mean that not all residents of a large household will mix and spread infections to other residents equally. However, in our study, which included seven different countries, it was not feasible to employ a stricter definition of household. We did not find any published data on people per room in our seven countries to facilitate a direct comparison, but a publication from The Gambia (another country in the meningitis belt) found lower levels of crowding [1].

The prevalence of self-reported respiratory symptoms was high, with nearly half of our study population (45.5%) reporting at least one respiratory symptom in the previous week. Although these symptoms are non-specific, self-reported and not validated, we considered that viral respiratory infections were a likely cause. The period of the study was however outside of the main period for influenza transmission [25]. Younger children, aged less than 5 years, were more likely to have symptoms than older children and adults. Despite higher levels of mixing amongst adults, the lower prevalence of symptoms in adults may be due to a shorter duration of symptoms or reduced susceptibility due to immunity.

People living in urban areas were less likely to report symptoms than those living in rural areas. We might have expected more respiratory illness in the urban area since people living there are more likely to mix frequently which is strongly associated with higher prevalence of symptoms. However, other factors which we did not measure, such as household income, may be important. Another study in Indonesia also found that respiratory disease (specifically respiratory syncytial virus lower respiratory tract infections in young children) was more common in rural areas compared to urban areas [26]. Particularly relevant for the MenAfriCar studies are the findings that respiratory tract infections may predispose to meningococcal disease [27], [28] and may be associated with meningococcal transmission [29].

Viral respiratory infections are highly transmissible which may explain the association with frequent mixing. Surprisingly our results show no association with household crowding or crowded sleeping arrangements. Crowding has previously been shown to be associated with increased rates [30] and severity of RSV [31] in The Gambia and Indonesia respectively. It is also an important risk factor for infections requiring prolonged or close contact for transmission, including tuberculosis [1], [32] and in developed countries, meningococcal disease [2], [33], [34]. It is possible that our findings have been influenced by our unrestrictive definition of a household (those who share a cooking pot).

Indoor smoke pollution is reported to increase the risk of acute respiratory infections [35], [36]. Our results show an increase in respiratory symptoms reported by people with kitchens outside or in a separate hut in a univariate analysis, but in the multivariate analysis we found no association between location of the kitchen and symptoms. We found no association between fuel and symptoms; this may be because the vast majority of study participants (88.5%) used wood or charcoal whereas the cleaner fuels (electricity, oil, paraffin, gas) were only used by 267 (1.5%). Cleaner cooking fuels have been found to reduce the risk of respiratory illness [36].

Other studies have highlighted the importance of population movements and mixing on disease transmission; coinciding with agricultural seasons [37], differences between men and women in Islamic society [38] and DNA fingerprint studies of TB transmission outside the household [39], [40]. This study quantifies social mixing patterns simply as attendance at different types of “social events or meeting places”. We did not measure more commonplace activities such as attending school or regular workplace, nor did we measure frequency, duration, repetition and clustering of contacts, e.g. by age or sex [41], [42]. These factors are likely to be important, so this is a key limitation of our study. The questions on social gatherings may have been more likely to be relevant to adults, so the amount of social contact in children in particular could have been underestimated. Nevertheless, our demonstration of considerable heterogeneity across this region suggests there is a need for more detailed studies of social contacts in meningitis belt countries. Future research could focus on collecting data on duration, type age and location of contacts using diary based methods [9] or recall at interview [10]. A detailed study of social contacts in a South African township showed that numbers of close contacts were 40% higher than in corresponding populations in industrialized countries [11]. This also illustrates the importance of generating geographically-specific measures of contact. The detailed social mixing pattern studies referenced above and others based on dynamics within households [43], [44] have contributed to our theoretical understanding of network analysis but the evidence from observational studies linking this with the impact on disease transmission is lacking.

Our study demonstrates the heterogeneity in crowding and social mixing across the African meningitis belt and finds that social mixing, but not household crowding, is associated with respiratory symptoms. This lays a foundation for further research into the impact of social mixing patterns on the spread of airborne infections through populations of sub-Sahara Africa.
